# Correction: Development of a non-infectious control for viral hemorrhagic fever PCR assays

**DOI:** 10.1371/journal.pntd.0013277

**Published:** 2025-07-09

**Authors:** Matthew A. Knox, Collette Bromhead, David TS Hayman

The Funding statement for this article is incorrect. The correct Funding statement is as follows: EBO-SURSY project funded by the European Union via the World Organisation for Animal Health (Grant 3000034275; OIE Laboratory (or Collaborating Centre) Twinning Project: Enhancing capacity for early detection of viral haemorrhagic fevers in Liberia through epidemiological and laboratory training).
