# Supplementary material for: Development of a non-infectious control for viral hemorrhagic fever PCR assays
Source: PLoS Negl Trop Dis. 2024 Apr 22;18(4):e0011390. doi: 10.1371/journal.pntd.0011390 (PMC11065202; doi:10.1371/journal.pntd.0011390)
Supplement: S1 Fig — Lanes are labelled as follows: A: 1kb ladder (expanded on right of figure), B: 5.5x10^12^ copies, C: 5.5x10^11^ copies, D: 5.5x10^9^ copies, E: 5.5x10^7^ copies, F: 5.5x10^5^ copies, G: 5.5x10^3^ copies, H: negative control. CCHFV: Crimean-Congo hemorrhagic fever virus, EBOV: Ebola virus, LV: Lassa virus, MARV: Marburg virus and RVFV: Rift Valley fever virus. (DOCX) [file pntd.0011390.s001.docx]

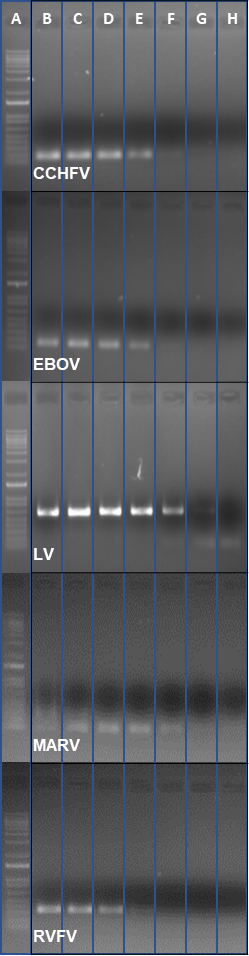

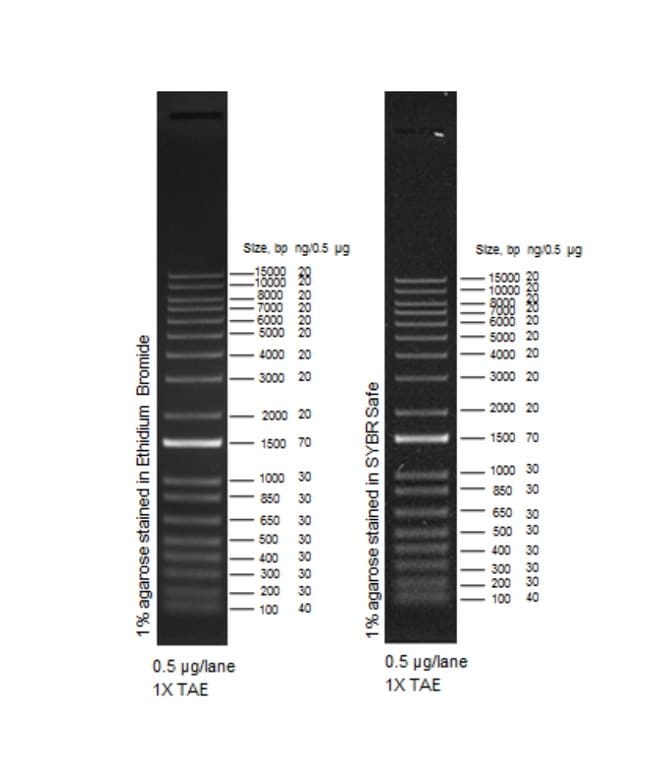


**Supplementary Figure 1.** RT-PCR gel results from dilution series experiment on five VHF virus assays. Lanes are labelled as follows: A: 1kb ladder (expanded on right of figure), B: 5.5x10^12^ copies, C: 5.5x10^11^ copies, D: 5.5x10^9^ copies, E: 5.5x10^7^ copies, F: 5.5x10^5^ copies, G: 5.5x10^3^ copies, H: negative control. CCHFV: Crimean-Congo hemorrhagic fever virus, EBOV: Ebola virus, LV: Lassa virus, MARV: Marburg virus and RVFV: Rift Valley fever virus*.*
