# Supplementary material for: Development of a non-infectious control for viral hemorrhagic fever PCR assays
Source: PLoS Negl Trop Dis. 2024 Apr 22;18(4):e0011390. doi: 10.1371/journal.pntd.0011390 (PMC11065202; doi:10.1371/journal.pntd.0011390)
Supplement: S1 Table — (DOCX) [file pntd.0011390.s002.docx]

**Supplementary Table 1**. Gel electrophoresis detection results for a dilution series experiment on five viral hemorrhagic fever virus assays.

| **Virus*** | **Dilution series** | **Neat** | **1:10** | **1:10^3^** | **1:10^5^** | **1:10^7^** | **1:10^9^** |
| --- | --- | --- | --- | --- | --- | --- | --- |
|  | **ng/mL** | **3.12E+02** | **3.12E+01** | **3.12E-01** | **3.12E-03** | **3.12E-05** | **3.16E-07** |
|  | **Copies/mL** | **5.53E+11** | **5.53E+10** | **5.53E+08** | **5.53E+06** | **5.53E+04** | **5.53E+02** |
| **CCHFV** | Positive | Positive | Positive | Positive | Positive | Weak positive | Negative |
| **EBOV** | Positive | Positive | Positive | Positive | Positive | Weak positive | Negative |
| **LV** | Positive | Positive | Positive | Positive | Positive | Positive | Weak positive |
| **MARV** | Positive | Positive | Positive | Positive | Positive | Positive | Weak positive |
| **RVFV** | Positive | Positive | Positive | Positive | Weak positive | Negative | Negative |

*CCHFV: Crimean-Congo hemorrhagic fever virus, EBOV: Ebola virus, LV: Lassa virus, MARV: Marburg virus and RVFV: Rift Valley fever virus.
