# Supplementary material for: Development of a non-infectious control for viral hemorrhagic fever PCR assays
Source: PLoS Negl Trop Dis. 2024 Apr 22;18(4):e0011390. doi: 10.1371/journal.pntd.0011390 (PMC11065202; doi:10.1371/journal.pntd.0011390)
Supplement: S2 Table — (DOCX) [file pntd.0011390.s003.docx]

**Supplementary Table 2**. Comparison of sample matrices (PBS v fecal) on RT-qPCR efficiency using the *Crimean-Congo hemorrhagic fever virus* (CCHFV) assay.

| **Copies/μL** | **Sample** | **1.0E+7** | **1.0E+6** | **1.0E+5** | **1.0E+4** | **1.0E+3** | **1.0E+2** | **1.0E+1** | **Neg**  **control** | **Pos control** |
| --- | --- | --- | --- | --- | --- | --- | --- | --- | --- | --- |
| **PBS** | **A** | 25.85 | 29.31 | 32.99 | 35.82 | 38.04 | N | N | N | 25.7 |
|  | **B** | 25.72 | 29.16 | 32.79 | 37.02 | N | N | N | N | 25.7 |
|  | **Mean Ct** | **25.79** | **29.24** | **32.89** | **36.42** | **38.04** |  |  |  |  |
| **Fecal** | **C** | 27.85 | 31.86 | 36.39 | 36.4 | N | N | N | N | 25.6 |
|  | **D** | 27.96 | 32 | 34.66 | 37 | N | N | 40.65 | N | 25.7 |
|  | **Mean Ct** | **27.91** | **31.93** | **35.53** | **36.7** |  |  |  |  | **25.7** |
