# Supplementary material for: Development of a non-infectious control for viral hemorrhagic fever PCR assays
Source: PLoS Negl Trop Dis. 2024 Apr 22;18(4):e0011390. doi: 10.1371/journal.pntd.0011390 (PMC11065202; doi:10.1371/journal.pntd.0011390)
Supplement: S3 Table — (DOCX) [file pntd.0011390.s004.docx]

**Supplementary Table 3**. Raw Cq values from time course experiments

| **Time** | **Cq value** | **EBOV** | **CCHFV** | **RVFV** | **Long LV** | **MARV** | **Short LV** |
| --- | --- | --- | --- | --- | --- | --- | --- |
| **Zero** | 1 | 29.99 | 29.05 | 29.7 | 31.7 | 27.8 | 32.05 |
|  | 2 | 30.27 | 29.1 | 30.27 | 31.75 | 27.27 | 32.41 |
|  | **Average** | **30.13** | **29.075** | **29.985** | **31.725** | **27.535** | **32.23** |
| **24 hours** | 1 | 33.46 | 31.53 | 33.97 | 34.4 | 30.09 | 34.15 |
|  | 2 | 32.92 | 31.20 | 33.72 | 34.38 | 29.59 | 34.04 |
|  | **Average** | **33.19** | **31.365** | **33.845** | **34.39** | **29.84** | **34.09** |
| **72 hours** | 1 | 33.81 | 32.58 | 34.42 | 37.19 | 30.25 | 36.71 |
|  | 2 | 33.76 | 32.54 | 33.97 | 38.18 | 30.26 | 37.6 |
|  | **Average** | **33.8** | **32.6** | **34.2** | **37.7** | **30.3** | **37.2** |
| **1 week** | 1 | 43.13 | 36.04 | 37.74 | 38.49 | 32.16 | 38.64 |
|  | 2 | 35.51 | 35.90 | 37.86 | N | 31.92 | 38.58 |
|  | **Average** | **39.32** | **35.97** | **37.8** | **38.49** | **32.04** | **38.61** |

EBOV: Ebola virus*,* CCHFV: Crimean-Congo hemorrhagic fever virus*,* RVFV: Rift Valley fever virus*,* LV: Lassa virus and MARV: Marburg virus*.*
