# Supplementary material for: Development of a non-infectious control for viral hemorrhagic fever PCR assays
Source: PLoS Negl Trop Dis. 2024 Apr 22;18(4):e0011390. doi: 10.1371/journal.pntd.0011390 (PMC11065202; doi:10.1371/journal.pntd.0011390)
Supplement: Attachment — Submitted filename: Response_12_12_23.docx [file pntd.0011390.s005.docx]

**Reviewer #1:** In their manuscript, Knox and colleagues present the development of synthetic DNA oligonucleotide sequences encoding primer binding sites of different viral haemorrhagic fever (VHF) viruses and their application in conventional and real-time RT-PCRs with the aim of implementing this strategy under field conditions. Generally, the development of in-vitro transcribed RNA as controls for various PCR assays is not entirely new. However, the general idea to establish protocols that support capacity building in areas where access to diagnostic laboratory services has been limited, is crucial to strengthen the laboratory infrastructure of the respective countries. Moreover, this study underlines the importance of epidemiological surveillance studies in these areas and the need for reliable diagnostics and well-trained personnel.

**Thanks**

Although the general performance of the in-vitro transcribed RNA seems promising, there are some shortcomings in the overall methodology that should be addressed. The description of some important points was insufficient or completely missing.

**Thanks, we have tried to address your concerns below.**

Some major points:
 p. 8, lines 89 ff.: Taxonomy. It seems that there is no unified way of using the virus taxonomy. Some viruses are written in italics, others not. For some, the species name was used (CCHF orthonairovirus and RVF phlebovirus). Here, one could go for the virus name like in Ebola, Lassa and Marburg virus. Only when referring to the species/genus/family, the name is written in italics. “[…] order Bunyavirales (italics!): Crimean-Congo haemorrhagic fever virus (non-italics!) (CCHFV; Crimean-Congo haemorrhagic fever orthonairovirus (italics!), family Nairoviridae (italics!), […]” and so on. It starts in the abstract and is continued in the Methods section.

**We thank the reviewer for this comment. We followed the ICVT correct terminology throughout and tried to minimize switching between species and virus throughout the document when we can. We have introduced the viruses, including the Latin species names and have used established ICVT virus names and abbreviations throughout the paper for clarity.**

p. 8, line 85: how randomly generated is this sequence between the primer binding sites? Is there anything to consider when generating it (e.g. RNA secondary structure, anything that could hamper RNA transcription)? How are the melting peaks affected by sequence design?

**These are all excellent points.**

**We used the random DNA generator from bioinformatics.org** [**https://www.bioinformatics.org/sms2/random_dna.html**](https://www.bioinformatics.org/sms2/random_dna.html) **to generate the 1000 bp sequence, then replaced bases at the primer binding sites. We did not check for RNA secondary structure and this may be an issue to consider in future studies, but since our assay was successful it does not appear to be an issue in our work.**

**The multi-assay design of our product did not allow for incorporation of realistic target sequences in between primers and the reviewer is correct that melting peaks are altered by the random sequence used in our design. However, future work might use a linear design, i.e. no overlap between amplicons which would allow for a realistic melt peak analysis as well as probe based qRT-PCR. That was not the purpose of this work though.**

**We have included text on all these issues in the discussion now (lines 268-270, 278-284), as they are good points.**

p. 8, line 87: the fragment contains primer binding positions. What about using probes in addition? Conventional PCR and melting curve analyses-based qPCR requires sequencing to avoid misinterpretation/to exclude contamination. Certainly, this can also happen in probe-based qPCRs. However, under field settings, sequencing may be a challenge. Also, melting curve analyses require intensive training of personnel. At the very least, please discuss advantages and disadvantages of all these methods.

**We thank the reviewer for these insightful thoughts and have added some additional material to the discussion (lines 284-286).**

p.10, line 109: does manufacturer’s instruction include DNAse digest? It would be helpful to mention it and discuss its importance when discussing the results.

**Thanks, again, this is a useful comment. Yes, this optional step was performed and has been added to the methods (line 116).**

When setting up all the assays, have PCRs been performed without reverse transcription? Was there an amplification?

**Not with all the assays in this study, but we did demonstrate this previously using positive control material for use in DNA based assays. We verified the sequence identity of the insert by amplifying with M13 primers. This is clarified in the text (line 100-101 in methods), but the details are not given and these PCRs were simply used to check the sequences were correct.**

How did the authors determine purity of RNA?

**We did not. This was not tested as the assay was originally designed for training purposes and use with lab reagents likely free of contaminants. In different settings this would be tested using nanodrop, but our assays and controls worked well in the complex fecal matrix we used in response to the reviewer comments. We added a comment on this in the discussion (lines 274-277).**

**Reviewer #2:** Most clinical laboratories in VHF-prone countries in sub-Saharan Africa use real-time PCR to diagnose EBOV and LASV infection. What is the rationale for using analogue RT-PCR in this paper?

**The rationale was to develop a control for training purposes in a low resource, veterinary diagnostic setting to detect the viruses that cause these zoonotic diseases. We have added text to the introduction to make this point more clearly (lines 68-73). As such, the analogue RT-PCR will be of value for any lab that has a thermocycler and gel electrophoresis equipment and for which small batches of reagents may be cheaper. However, our assay can also be used in quantitative analyses and that is the focus of the text, as this RT-qPCR tells us more about the utility of our approach than RT-PCR. We explore the application of the assay for real-time PCR to cover potential clinical applications and have added material to the discussion exploring other potential applications using similar synthetically designed control material (lines 274-284).**

**Reviewer #3**: In this manuscript Knox et al describe their development of a non-infectious control for viral hemorrhagic fever PCR assays. The article is well-written and results are clearly presented. Overall the idea and preliminary results with the control(s) appear promising. However, the authors overstate or misunderstand the application of the controls in their current state of development. Are the controls for use in the field or a molecular biology lab? If the former, then how do you operate a quality-equivalent thermal cycler in an area where "access to refrigeration and cold storage is often not possible"?. If the latter, then why is so much emphasis put in the manuscript about low-resource settings? The control as currently presented/validated is more appropriate for developed world diagnostic laboratories where indeed lab staff from low-resource countries could be trained.

**We thank the reviewer for these comments and have clarified the intended setting for the use of the described control assay (lines 68-73). To clarify, we originally designed the assay for training purposes in Leon Quist Ledlum Central Veterinary Diagnostic Laboratory, Liberia as part of a WOAH (then OIE) twinning program to train personnel there in surveillance methods for VHFs, but the assay could easily be used in similar settings elsewhere, for example in veterinary settings primarily to detect the presence of these viruses diseases in animal populations where labs may not be as well-resourced as the clinical labs, along with these being safe, reproducible, and flexible positive controls that reduce the need to source target RNA from reference laboratories, which is often limited.**

Scientifically, the biggest concern I see with the control validation is the fact that experiments are always being performed in sterile PBS, sterile water, PCR grade water, and RNase-free water. Minimally the authors should present results with fresh blood samples spiked with the controls. Ideally the control would be encapsulated with a pseudovirus or proteinaceous shell.

**Thanks, these are both excellent points. We have revised the text to emphasize the purpose of the work (see above). While the control is intended to work as a training tool and for reagent viability we recognize the reviewers point and have added analyses and the necessary text demonstrating the performance of the assay in a realistic matrix. We spiked a fecal sample with control material and ran the CCHFV RT-qPCR alongside PBS counterparts over a dilution series from 1E7 to 10 copies/μL in 10-fold dilutions, in duplicate. These results, which showed analogous assay performance at the LOD, are now presented in the manuscript (lines 216-220).**
Another problem with the validation is the idea that 28C is an adequate challenge for RNA for these VHFs, many of which occur in the middle east, Sahara, West Africa, and equatorial Africa where average daily temperatures are around 27C.

**Our intention was to demonstrate that the assay would perform outside of ideal laboratory conditions. Yes, we could have tested performance of all reagents more and over a greater range, but further testing would be required depending on the precise conditions elsewhere. However, above these temperatures other issues relating to the assay reagents would also become a problem for these laboratories, so we have assumed some infrastructure is available.**

Some minor comments:
Line 41: is "but" or "and" more appropriate?
Line 51: change animal to animals.
Lines 53-56: Need reference for this statement.
Line 58: This sort of "must" language should be removed from scientific publication.

**Thanks. We have changed or amended all of the above minor points.**

**Reviewer #1:** Some major points:
p. 13, line 169 ff.: the detection limit is around 5 million copies per µl for some of the conventional PCRs, that seems a lot. What about the dilutions of 1:10^4, 1:10^6 and 1:10^8? Have those been performed and tested? It would give more accuracy to the detection limit and maybe even improve performance of the PCRs. Especially when comparing it to the qPCR data where 50 copies were sufficient. This is round about 5 log. Have the authors tested the effect different primer concentrations may have? That would be important to know at this point.

**It is common that real-time versions of conventional presence/absence amplification assays demonstrate increases in sensitivity of several logs. We only tested the performance of the conventional PCR over the range and dilutions which were presented and agree that the extra dilutions suggested would give better resolution. However, in our view the material we have presented gives the necessary information i.e. the approximate minimum copy numbers to be able to view a band on a gel for each assay. Since it is a positive control, this is sufficient information for use in training or for surveillance studies. Future studies may use the control for further optimization of primer concentrations and indeed, other parameters in the PCR but this was not the intention of this study.**

For the supplementary Figure 1, it would help to have the DNA ladder size indicated, maybe at least for some of the bands.

**We agree. We have added this information to the SI figure.**

p. 13, line 180: Table 3: the optimal annealing temperature has a broad range for almost all the assays. How was this determined? Please describe. What were the quality criteria to consider the temperature optimal? P. 14, l. 192, please mention the criteria to decide for 56°C annealing temperature.

**The LightCycler 96 has a gradient block and we used this to run each of the assays in duplicate in two sets of temperature gradients: 55°C-62°C, then 62°C to 65°C. This is described in lines 158-162.**

**Here is an example of the cycling conditions on the LightCycler for a temperature gradient assay:**

**Cycling conditions**:

| **Programme** | **cDNA synthesis** | **Denaturation** | **Amplification** | | | **Melt** | | | | **Cooling** |
| --- | --- | --- | --- | --- | --- | --- | --- | --- | --- | --- |
| Cycles | 1 | 1 | 55 | | | 1 | | | | 1 |
| Analysis Mode | None | None | Quantification | | | HRM | | | | None |
| Segment number | 1 | 1 | 1 | 2* | 3 | 1 | 2 | 3 | 4 | 1 |
| Target Temp (°C) | 50 | 95 | 95 | Gradient 55-62 across 12 tubes | 72 | 95 | 40 | 50 | 98 | 37 |
| Inc Time (sec) | 600 | 180 | 5 | 15 | 30 | 60 | 60 | 1 | 0 | 30 |
| Temp trans rate (°C/sec) | 4.4 | 4.4 | 4.4 | 2.2 | 4.4 | 4.4 | 2.2 | 1 | 0.07 (15 readings per degree) | 2.2 |
| Acquisition Mode | None | None | None | None | Single | None | None | None | Cont | None |

**Sample layout showing temperature changes from 55°C to 62°C as an example:**

|  | **1** | **2** | **3** | **4** | **5** | **6** | **7** | **8** | **9** | **10** | **11** | **12** |
| --- | --- | --- | --- | --- | --- | --- | --- | --- | --- | --- | --- | --- |
| **Row A** | RVF  55C | RVF 55.4C | RVF 56C | RVF 56.8C | RVF 57.8C | RVF 58.7C | RVF 59.7C | RVF 60.5C | RVF 61.2C | RVF 61.7C | RVF  62C | RVF  62C |
| **Row B**  **dup** | RVF  55C | RVF 55.4C | RVF 56C | RVF 56.8C | RVF 57.8C | RVF 58.7C | RVF 59.7C | RVF 60.5C | RVF 61.2C | RVF 61.7C | RVF  62C | RVF  62C |
| **Row C** | CCHF 55C | CCHF 55.4C | CCHF 56C | CCHF 56.8C | CCHF 57.8C | CCHF 58.7C | CCHF 59.7C | CCHF 60.5C | CCHF 61.2C | CCHF 61.7C | CCHF 62C | CCHF 62C |
| **Row D**  **dup** | CCHF 55C | CCHF 55.4C | CCHF 56C | CCHF 56.8C | CCHF 57.8C | CCHF 58.7C | CCHF 59.7C | CCHF 60.5C | CCHF 61.2C | CCHF 61.7C | CCHF 62C | CCHF 62C neg |
| **Row E** | MARV 55C | MARV 55.4C | MARV 56C | MARV 56.8C | MARV 57.8C | MARV 58.7C | MARV 59.7C | MARV 60.5C | MARV 61.2C | MARV 61.7C | MARV 62C | MARV 62C |
| **Row F**  **dup** | MARV 55C | MARV 55.4C | MARV 56C | MARV 56.8C | MARV 57.8C | MARV 58.7C | MARV 59.7C | MARV 60.5C | MARV 61.2C | MARV 61.7C | MARV 62C | MARV 62C |

**The average Cq value of each duplicate was compared to find the optimal range of annealing temperatures. This, with explanation of the Cq, is added to lines 163-165.**

p. 14, line 184: Figure 3, if the synthetic construct does not include the authentic sequence amplified (but only a random sequence as mentioned above), how does that affect the melting peaks when comparing it with that of an (authentic) field sequence? Please include at least a statement in your discussion.

**Thanks, yes, this is an issues (see previous text) and we have acknowledged this in the discussion (lines 278-286)**

p. 15, line 208: please add abbreviations to the figure legend. What does Cq mean, what is displayed at all? The figure legend is a little short in information.

**We agree and have revised the figure caption accordingly.**

p.16, line 229 ff., please also discuss the capacity building needed to train personnel in designing a synthetic construct (and possibly the difficulty to obtain synthesized sequences in some low to middle income countries).

**In this case we designed the synthetic construct ourselves but can certainly see the value of training end-users to design their own controls to meet specific project needs. There are many barriers to engaging learners from LMICs, which is the topic of another manuscript in preparation. However, we have added text explicitly addressing this (lines 265-267). We have also added a point that the synthetic control arrives in dried form and is relatively stable until reconstituted (lines 112-114).**

**Reviewer #2**: RT-PCR and RT-qPCR

•	The authors have not demonstrated the relevance of these RT-PCR and RT-qPCR conditions by showing that they can successfully amplify nucleic acids from any of the five different VHF pathogens in real or spiked samples.

**Thanks, this is an important point. While the control is intended to work as a training tool and for reagent viability we recognize the reviewers point and have added material demonstrating the performance of the assay in a realistic matrix. We spiked a fecal sample with control material and ran the CCHFV RT-qPCR alongside PBS counterparts over a dilution series from 1E7 to 10 copies/μL in 10-fold steps. These results, which showed analogous assay performance at the LOD, showing our assays and controls work well in matrices like feces, whose complex composition make the presence of PCR inhibitors more likely than many types of test matrix, and are now presented in the manuscript in the methods, results and discussion.**

Stability of PCR reagents

•	This section is poorly designed.
•	Why are the authors testing the stability ToughMix and Eva Green reagents, when the focus of the paper is on the synthetic positive control material? One of the main reasons that diagnostic PCR reactions fail in the field is due to degradation of the positive control nucleic acids. What the authors should have tested is the stability of their synthetic control material, at different temperature, through different number of freeze-thaw cycles.

**This is a good point. Of course, a point of our project is to provide a stable, reproducible, non-infectious control. Our plasmid vector is stored at -20°C to – 80°C and will be stable for years, as shown in other studies [*Matange, K., Tuck, J.M. & Keung, A.J. DNA stability: a central design consideration for DNA data storage systems. Nat Commun 12, 1358 (2021). https://doi.org/10.1038/s41467-021-21587-5*]. We performed amplification of stored RNA for the feces matrix study, as requested by the reviewers and found it to perform similarly to before but with a reduction in sensitivity demonstrating that long term storage or RNA template and freeze thaw cycles will affect assay sensitivity (lines 221-223). This was performed over year since the first experiments.**

**The transcribed RNA will degrade just like any other RNA and so will need storage like any other RNA, though a key point of our work is that we have generated a DNA-plasmid based control that can be utilized to generate single strand RNA effectively as and when a researcher or diagnostician requires it. Further work would be needed to determine how stable the plasmid vector is and how susceptible it is to freeze thaw cycles. We have not done that in the time available, but added this as a comment to the discussion (lines 272-278).**

•	The ambient temperature used (12-28°C) is inadequate. The ambient temperature in West Africa can reach 38°C in the dry season.

**This is also a good point, however, the reagents required to perform the assays themselves require the components to be stored in (ideally) a constant temperature freezer at -25°C to -15°C and protected from light. Our intention was to demonstrate that the assay would perform outside of ideal laboratory conditions, but further testing would be required depending on the precise conditions elsewhere. We have to assume that there is some infrastructure for the work for now, so hope that this suffices.**

•	There is no control. Would the Cq also increase after 7 days if the reagents were stored at 2-8°C or -20°C?

**Thanks, this is also a fair point, except that we know that if we store reagents as per the manufacturers recommendations the reagents are stable, and the reagents at time zero in our experiment have been stored for many weeks at the recommended conditions. We acknowledge a control would be useful here for illustrative purposes. We have added comments addressing this as per above.**

**Reviewer #3:** (No Response)

**Conclusions**
 *-Are the conclusions supported by the data presented? -Are the limitations of analysis clearly described? -Do the authors discuss how these data can be helpful to advance our understanding of the topic under study?-Is public health relevance addressed?*

**Reviewer #1:** see above suggestions concerning the improvement of the discussion.

**Thanks. We have addressed the comments above. We hope this satisfies the reviewer and editor.**

**Reviewer #2:** The study is poorly designed and lacks key controls.

The results do not fully demonstrate relevance or application.

**We hope that the revisions now satisfy this reviewer. We have tried to be more clear in the text and clarify what the purpose of the study was by including relevant background information on the goals of the twinning project and the work, along with additional work that hopefully the reviewer will see as valuable.**

**Reviewer #3:** (No Response)

*Editorial and Data Presentation Modifications? Use this section for editorial suggestions as well as relatively minor modifications of existing data that would enhance clarity. If the only modifications needed are minor and/or editorial, you may wish to recommend “Minor Revision” or “Accept”.*

**Reviewer #1:** (No Response)

**Reviewer #2**: Line 92
The correct abbreviation for Ebola virus is EBOV.

Line 93
The correct abbreviation for Marburg virus is MARV.

**We have made these revisions.**

**Reviewer #3**: (No Response)

*Summary and General Comments: Use this section to provide overall comments, discuss strengths/weaknesses of the study, novelty, significance, general execution and scholarship. You may also include additional comments for the author, including concerns about dual publication, research ethics, or publication ethics. If requesting major revision, please articulate the new experiments that are needed.*

**Reviewer #1**: try Taqman-based/probe-based PCR in comparison with conventional and melting curve-based PCR.

**We have not done this. This would require us to redesign of the synthetic DNA to allow incorporation of probes, design new probes, and add additional costs, because probes are neither cheap, nor very stable. We feel this is another separate study beyond what we aimed. We hope our revisions mean that the purpose of the work is now clearer.**

complete dilution series (10^4/6/8 were missing)

**There is a complete dilution series for the RT-qPCR, with a limit of detection of 50 copies per μL. We do not think this level of detail is needed, given the purpose of the work, particularly for the RT-PCR.**

show experiments to determine annealing temperature

**Please see the above reviewer response. We have clarified the text in the methods to explain this process too.**
  **Reviewer #2**: The development of non-infectious control materials for VHF diagnosis is welcome. But the result presented in this paper is not complete. The experimental design for reagent stability is flawed. The use of the invented reagent has not been fully validated.

**We have revised the text. Our intention is not to provide a fully validated assay. Our work is showing the development of a non-infectious control for viral hemorrhagic fever PCR assays, as our manuscript title states, and we hope the revisions and additional work, such as testing the assays and control material in feces, a natural complex matrix, demonstrate the applicability of the method for use as a positive control for a range of applications.**

**Reviewer #3:** (No Response)
