# Supplementary material for: Development of a non-infectious control for viral hemorrhagic fever PCR assays
Source: PLoS Negl Trop Dis. 2024 Apr 22;18(4):e0011390. doi: 10.1371/journal.pntd.0011390 (PMC11065202; doi:10.1371/journal.pntd.0011390)
Supplement: Attachment — Submitted filename: PNTD-D-23-00631R1.docx [file pntd.0011390.s006.docx]

**Reviewer's Responses to Questions**

**Methods**

Reviewer #4: In the manuscript, the authors present the development of synthetic DNA, infectious control for the viral hemorrhagic fevers PCR assays, this is a good subject, as for a lot of viral infections it is hard to extract the positive controls, particularly where the access to the source material is problematic or requires high level biosafety containment.

We thank the reviewer for this comment and are pleased to see our modifications to the manuscript have made the purpose of the study clearer.

there are some grammatical errors in line31 and needs to be changed to "are". the same in line62.

We have checked the text and corrected these errors.

Reviewer #5: The study only evaluated fecal samples, but should include other sample types unless other parts of the manuscript are updated to clearly reflect this is only one sample type.

We have added a sentence to the discussion (**lines 279-282**) stating that we have only tested this in one sample type. Faeces are arguably the most challenging potential sample matrix, which is why we chose it, but we do now acknowledge that further testing may be advisable in different sample types e.g. blood, urine etc.

The authors mention that the lyophilized plasmid DNA could be stored in the relatively stable dried form, but include no experiments that indicate for how long and at what temps it can be stored and still successfully used in this assay. This is important given they highlight that this is a good tool for use in LMICs.

Rather than re-running the experiments with stored lyophilised plasmid DNA would be expensive and time consuming. The source company has provided information on storage conditions, and we now include information on the recommended storage conditions at a range of temperatures for lyophilised plasmid DNA (**line 119**).


**Results**

Reviewer #4: The authors have not mentioned the importance of Rt PCR and RT qPCR.

We have added a short introductory statement (**lines 77-81**) about the importance of these methods to the introduction, noting that PCR based approaches for clinical diagnosis, research and surveillance are relevant despite the increasing use of rapid diagnostic tests, which typically have lower sensitivity and specificity compared to PCR, and are host-species agnostic.

We refer the reader to Muzembo BA, Kitahara K, Ohno A, Ntontolo NP, Ngatu NR, Okamoto K, Miyoshi SI. Rapid diagnostic tests versus RT-PCR for Ebola virus infections: a systematic review and meta-analysis. Bull World Health Organ. 2022 Jul 1;100(7):447-458. doi: 10.2471/BLT.21.287496. Epub 2022 Jun 1. PMID: 35813519; PMCID: PMC9243686 for the evidence of this.

the figures are of sufficient quality.

We thank the reviewer for this comment.

Reviewer #5: Figures images are low quality and difficult to read but content is valuable.

Prior to resubmission all figures were processed through the PLOS NTD PACE program and met the required standards. However, if needed we can resubmit.


 **Conclusions**

Reviewer #4: (No Response)
Reviewer #5: Limitations of testing only one sample type should be further discussed.

We have added a sentence to the discussion (**lines 279-281**) – see previous comment from R5.

Public health relevance is well addressed.

We thank the reviewer for this comment.

Clarity edits are needed.

We thank the reviewer for this comment, and we have checked the manuscript for clarity throughout and made minor edits where necessary.

**Editorial and Data Presentation Modifications**

Reviewer #4: (No Response)

Reviewer #5: General clarity edits are necessary. The text can be modified to be clearer and more direct.

We thank the reviewer for this comment, and we have checked the manuscript for clarity throughout and made minor edits where necessary.

**Summary and General Comments**

Reviewer #4: the paper has a good relevance but needs some minor clarifications and modifications.

We thank the reviewer for this comment, and we have checked the manuscript for clarity throughout and made minor edits where necessary.

Reviewer #5: The presented technology is valuable and useful, but the analyses of its use lacks important controls (alternate sample types, additional temperature storage testing) that need to be addressed before this can be presented as widely applicable controls.

We thank the reviewer for this comment and are glad that they see the useful applications of the study. Regarding testing in other matrices and storage of lyophilised product, we have added additional content to the revised manuscript (see previous comments for details).
